# A Treatise on the Diseases of the Eye

**Published:** 1882-06

**Authors:** 


**A Treatise on the Diseases of the Eye.** By HENRY D. NOYES, A. M., M. D. New York: William Wood & Co.

The publishers of the Library of Standard Medical Authors, made a fortunate selection in inviting the writer of this volume to present a digest of the latest views in ophthalmology. From the very first chapter it is evident that a teacher of experince is dealing with his subject from a "standpoint that is clinical and with a purpose that is practical." Moreover, one who remembers former papers and monographs of the writer, recognizes here the tendency to linger in fields which he was want to cultivate, and beholds familiar figures which have also done good service elsewhere. Let it not be understood that this is counted as a disadvantage, for the book is written more for the student than for those acquainted with ophthalmological literature, and as such, it is on the whole, an eminent success. One might criticise the arrangement of the subject matter, or differ from the writer as to statements concerning a few minor points, but where an opinion must be summed up in the few words of a short notice like this, it can only be one that is favorable in the extreme. Studends will find here a concise and clear exposition of many a point over which they have been puzzled, and more than one practitioner will turn to this book to seek for guidance in the perplexities of some obstinate "eye case."

